# The Homeobox BcHOX8 Gene in Botrytis Cinerea Regulates Vegetative Growth and Morphology

**DOI:** 10.1371/journal.pone.0048134

**Published:** 2012-10-25

**Authors:** Zsuzsanna Antal, Christine Rascle, Agnès Cimerman, Muriel Viaud, Geneviève Billon-Grand, Mathias Choquer, Christophe Bruel

**Affiliations:** 1 Unité Mixte de Recherche 5240 - Microbiologie, Adaptation et Pathogénie; Université Lyon 1, CNRS, Bayer CropScience, Villeurbanne, France; 2 Biologie et Gestion des Risques en Agriculture - Champignons Pathogènes des Plantes, INRA 1290, Thiverval-Grignon, France; University of Wisconsin - Madison, United States of America

## Abstract

Filamentous growth and the capacity at producing conidia are two critical aspects of most fungal life cycles, including that of many plant or animal pathogens. Here, we report on the identification of a homeobox transcription factor encoding gene that plays a role in these two particular aspects of the development of the phytopathogenic fungus Botrytis cinerea. Deletion of the BcHOX8 gene in both the B. cinerea B05-10 and T4 strains causes similar phenotypes, among which a curved, arabesque-like, hyphal growth on hydrophobic surfaces; the mutants were hence named Arabesque. Expression of the BcHOX8 gene is higher in conidia and infection cushions than in developing appressorium or mycelium. In the Arabesque mutants, colony growth rate is reduced and abnormal infection cushions are produced. Asexual reproduction is also affected with abnormal conidiophore being formed, strongly reduced conidia production and dramatic changes in conidial morphology. Finally, the mutation affects the fungus ability to efficiently colonize different host plants. Analysis of the B. cinerea genome shows that BcHOX8 is one member of a nine putative homeobox genes family. Available gene expression data suggest that these genes are functional and sequence comparisons indicate that two of them would be specific to B. cinerea and its close relative Sclerotinia sclerotiorum.

## Introduction

Botrytis cinerea is the causal agent of grey mould on grapes, strawberries and hundreds of other dicot plants [1]. Infection by this ascomycetous necrotrophic fungus usually begins with landing and attachment of asexual spores (conidia) on the host surface. Following germination and production of a germ tube, penetration of the plant tissues occurs via the development of a single-cell appressorium-like structure or that of mycelium carrying multi-cellular ‘infection cushions’ [2]. Entrance into the plant is not thought to rely on mechanical breaking of its barrier, but would rather depend on the secretion of a large panel of lytic enzymes and toxic metabolites [3]. The killing and degradation of the plant cells allows feeding and growth of the fungal hyphae, the formation of primary lesions and, later, invasion and complete maceration of the host tissues. Production of new conidia in large numbers eventually completes the fungus life cycle. Survival of B. cinerea in the environment is increased through the production of resistance structures called sclerotia. Under appropriate conditions, these highly melanized structures can produce new mycelium or, in the presence of micro-conidia of opposite mating type, sexual organs called apothecia from which sexual spores (ascospores) are released. As an opportunistic pathogen, B. cinerea is able to complete its life cycle on both decaying and living plants.

In the last decade, molecular tools adapted to B. cinerea developed [4]–[7] and molecular descriptions of its biology expanded. The role of selected enzymes [8]–[16], metabolites [17]–[21], transporters [22], [23], stress response elements [24], [25], cell wall building enzymes [26], [27] or signalling pathways [28]–[39] was hence revealed or clarified, most times in relation to plant infection. Moreover, the recent release and analysis of the fungus genome sequence [40] has added tremendous force to the task of understanding the necrotrophic plant-fungus interaction, and first outcomes of broader studies have emerged [3], [16], [41]–[43].

Modulation in the expression of specific subsets of genes is acknowledged to play a central role in cell adaptation to new environmental conditions, as well as in cell differentiation. Gene expression is either repressed or activated in response to physiological or environmental stimuli, and this is orchestrated by the cell transcription factors acting as targets of the cellular signalling network. Within this framework, and in the case of fungal diseases, transcription activators and repressors are therefore expected to control most of the transitions between the described infection stages, and this is likely to also apply to plant invasion by B. cinerea. A total of 419 transcription factors have been predicted from the genome sequence of this fungus [40] and most about them remains to be characterized since only a few have been studied [39], [44]–[48].

Master regulators of development that were first discovered in the fly Drosophila melanogaster are the «homeotic» or «homeobox» genes [49]. These genes contain a 180-bp DNA sequence called the homeobox whose translation leads to the production of a 60-bp DNA binding motif named the homeodomain. Homeobox genes are found in insects, animals, plants and fungi, and thousands of them have been identified that can be grouped into different classes [50]. These genes have been shown to play major roles in developmental processes such as differentiation and reproduction. In fungi, only few homeobox genes have been described, but most of them are involved in hyphal growth, sexual development, appressorium formation or either conidia or microconidia production [51]–[64].

In B. cinerea, homeobox genes have not attracted much attention so far, but one has been characterized in the context of a MAP-kinase cascade study [39]. The absence of Ste12 leads to a reduction in growth rate, the formation of dark aggregates in colonies grown on solid media, a partial defect in sclerotia formation, the formation of abnormal and non-functional appressoria, a delayed infection and a slower plant tissues colonization. In the present study, we identify the putative homeobox gene family in B. cinerea and we report on the functional characterization of its eighth member.

## Results

### Prediction of a Homeobox Gene Family in B. Cinerea

Fungal transcription factor sequences have been analyzed by Park et al. and compiled in the FTFD database [65]. Homeobox genes constitute one distinct family in which eleven members are predicted in B. cinerea out of the 419 transcription factors predicted in this fungus [40]. As shown in Table I, eight of these genes are identified by the homeodomain InterPro term IPR001356 (found in 1238 fungal sequences - http://www.ebi.ac.uk/interpro/). BLAST sequence analysis supported the homeobox prediction for 6 of these genes since homologues with predicted homeobox function were found (E-values: e^−23^ to e^−144^). BLAST results for genes 4 and 5 revealed only one homologue with unknown function in Sclerotinia sclerotiorum, the closest relative of B. cinerea. All eight genes segregate into eight different homeobox clades according to the phylogenic analysis performed by Park et al. [65], and this suggests functional specificity of the corresponding homeodomain proteins [66]. These eight genes were named BcHOX1 to BcHOX8 and orthologues to all were found in S. sclerotiorum, as determined by First BLAST Hit analysis (BcHOX1 and 4), BLAST Bi-Directional Best Hit analysis (BcHOX2-3,5-8) and verification of loci synteny conservation between the two fungal genomes. The presence of the IPR001356 homeodomain in all S. sclerotiorum orthologues was finally confirmed except for SsHOX4, for which a central 100 amino-acids region does not match its counterpart containing the homeodomain in BcHOX4 (Structural annotation of these two genes genomic sequences was verified by manual curation). Besides the BcHOX1-8 genes, one FTFD predicted homeobox gene (BC1G_10211.1) contains the STE-like IPR003120 term found in 183 fungal sequences (Table I). This gene is the yeast STE12 gene homologue in B. cinerea and has already been characterized [39]. At last, two genes (BC1G_05030.1, BC1G_11994.1) contain the homeobox-protein, antennapedia type, IPR001827 term (found in 5 fungal sequences). These genes are not found in the phylogenic analysis performed by Park et al. [65], and our BLAST sequence analysis did not support a homeobox transcription factor prediction; BC1G_11994.1 is predicted a tetracenomycin polyketide synthesis hydroxylase while all BC1G_05030.1 homologues are hypothetical proteins. These two genes were not further considered and the homeobox gene family would hence be constituted of nine members altogether.

**Table 1:** Predicted homeobox genes in B. cinerea**.**

Name	Gene number	Accession number	InterPro Domain	FTFD Phylogenetic Clade	Predicted protein length	S. sclerotiorum Orthologues	Conserved InterPro Domain	Detected ESTs	Expression data	
									fold change	Pvalue
BcHOX1	BC1G_15930.1bt4exctg_0160	XM_001545489	IPR001356	7	129 aa	SS1G_08681.1	yes	no	−3.1	6.3 10^−6^
BcHOX2	BC1G_06341.1BofuT4_P070100.1	XM_001555161		17	1422 aa1429 aa	SS1G_03098.1		yes	−1.54	0.004
BcHOX3	BC1G_04097.1BofuT4_P037030.1	XM_001556662		4	280 aa656 aa	SS1G_01567.1		yes	1.13	0.98
BcHOX4*	BC1G_09758.1BofuT4_P116730.1	XM_001551438		isolated	495 aa495 aa	SS1G_01322.1	no	no	−1.14	0.014
BcHOX5*	BC1G_12223.1BofuT4_P163050.1	XM_001548942		20	757 aa353 aa	SS1G_04303.1	yes	no	1.32	0.60
BcHOX6	BC1G_06476.1BofuT4_P051510.1	XM_001554903		10	465 aa322 aa	SS1G_00669.1		no	3.27	0.12
BcHOX7	BC1G_00385.1BofuT4_P046240.1	XM_001561250		14	135 aa598 aa	SS1G_04522.1		yes	1.35	0.23
BcHOX8	BC1G_10953.1BofuT4_P155900.1	XM_001550060		13	593 aa593 aa	SS1G_11635.1		yes	1.37	0.25
Ste12(BcHOX9)	BC1G_10211.1BofuT4_P086790.1	XM_001551335	IPR003120	15	679 aa396 aa	SS1G_07136.1	yes	yes	2.96	0.51

The putative homeobox genes are presented with their corresponding number in both the B05-10 (BC1G_) and T4 (BofuT4_) strains whose genomes are sequenced (in the T4 genome, BcHOX1 is found within the excluded contig bt4exctg_0160). Their phylogenetic clades is indicated as proposed by the fungal transcription factor database (FTFD). Orthologues found in S. sclerotiorum are indicated with the results of the InterPro domain conservation. Expression data are shown through recorded ESTs and transcriptomics results of sunflower infection (48 hours) compared to liquid cultures [40]. Asterisks label the two genes with no homologue found in other sequenced fungal genomes except S. sclerotiorum. BcHOX9 has already been characterized by Schamber *et al.* as the Ste12 gene [39].

Exploration of the B. cinerea genome database (http://urgi.versailles.inra.fr/Species/Botrytis) showed that ESTs have been collected for 5 of the 9 predicted BcHOX genes while the expression of all these genes has been observed in a comparative transcriptomics study (Table I). Besides, up-regulation (×2.28; Pvalue 10^−2.54^) of BcHOX8 during the course of Arabidopsis thaliana infection was reported by Gioti et al. [67]. Altogether, these expresssion data would indicate that the eight uncharacterized BcHOX1-8 genes are most likely functional genes.

### Functional Study of BcHOX8

**Figure 1:**
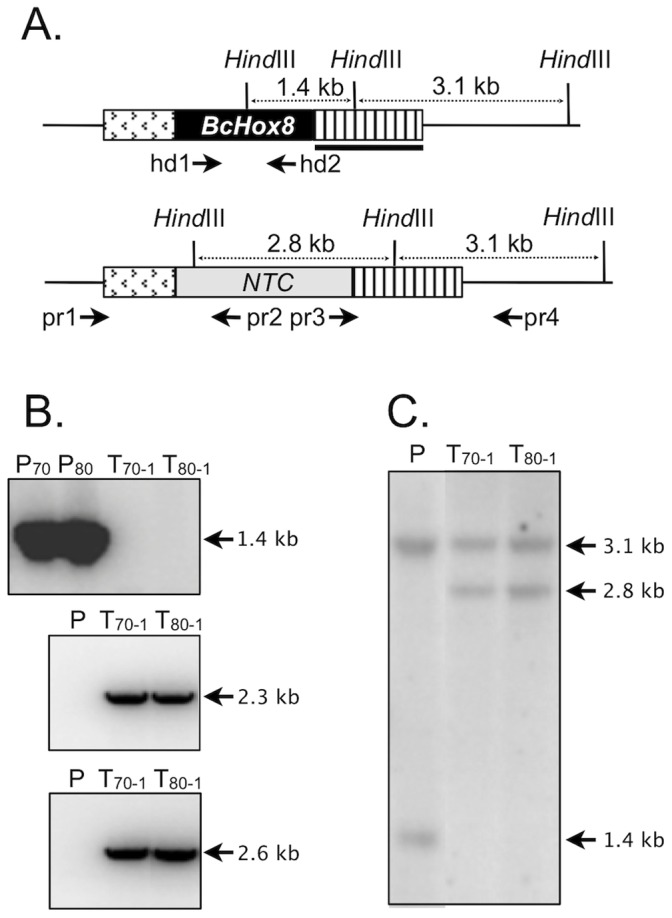
Deletion of the BcHOX8 gene. (A) Schematic representation of the BcHOX8 gene replacement by the nourseothricin (NTC) resistance gene flanked by 1.5 kb of downstream and 0.95 kb of upstream sequences from the BcHOX8 locus. Distances between HindIII restriction sites are shown with dotted double arrows. The probe used for Southern analysis and the primers used for PCR analysis are shown as a black bar and arrows, respectively. (B) PCR analysis of the parental (T4/Δku80 (P80) and B05-10/Δku70 (P70)) and transformed strains (T4-T_80-1_ (T_80-1_) and B05-T_70-1_ (T_70-1_)) using three primers (shown in A) combinations: hd1 and hd2 (top), pr1 and pr2 (middle) or pr3 and pr4 (bottom). (C) Southern blotting analysis of the parental T4/Δku80 strain (P) and one transformant of each genetic background (T4-T_80-1_ and B05-T_70-1_) using HindIII to digest genomic DNA and the DNA probe indicated in (A) to reveal the fragments.

With no experimental data available for any of the BcHOX gene except BcHOX8 (up-regulation during A. thaliana infection), the latter was chosen as the first target for functional analysis. We constructed a DNA fragment in which a nourseothricin (NTC) resistance cassette was flanked by upstream and downstream genomic sequences from the BcHOX8 locus (Fig. 1A). Since strains genetic backgrounds have been shown to influence the impact of single gene deletion in B. cinerea [68]–[69], we opted for the introduction of the BcHOX8 deletion cassette in two different strains and we chose the high-frequency-gene-replacement strains B05-10/Δku70 and T4/Δku80 previously constructed by Choquer et al. [6]. Two independent protoplast transformations were carried out that produced 1+6 B05-10/Δku70 and 1+5 T4/Δku80 nourseothricin-resistant transformants. The first transformant of each genetic background (B05-T_70-1_ and T4-T_80-1_) was purified by single-spore isolation and subjected to molecular analysis (Fig. 1B). The BcHOX8 gene could not be detected by PCR analysis in these strains while amplifications of the left and right junctions of the integrated exogenous DNA fragment could. As expected, opposite patterns were obtained for the two parental strains. Amplification of the resistance gene was positive in the transformants and negative in the parents (data not shown). These results were confirmed by Southern blotting since a hybridization profile compatible with a single gene replacement event and no ectopic DNA integration were revealed for each transformant (Fig. 1C). These two strains were selected for the rest of the study.

### The Arabesque Phenotype

**Figure 2:**
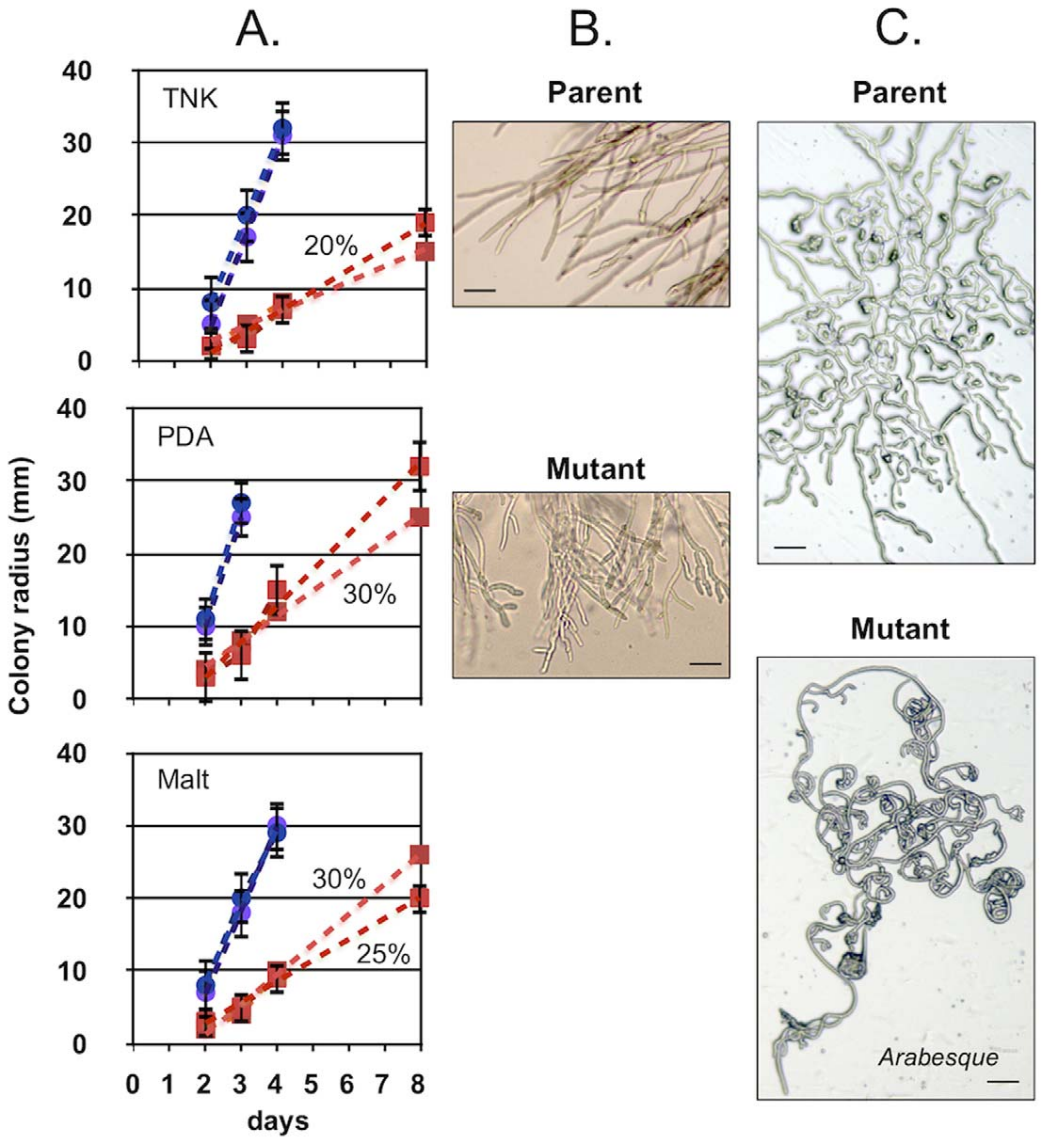
Impact of BcHOX8 deletion on the B. cinerea hyphal growth. (A) Colonies growth kinetics on TNK, PDA and Malt medium for the two parental strains (B05-10/Δku70 (empty circle) and T4/Δku80 (empty square)) and the two respective mutant strains (B05-T_70-1_ (filled circle) and T4-T_80-1_ (filled square)); 10 cm dishes were used. (B and C) Microscopic observations of the colonies edges (B) and the conidium-derived hyphal growth on hydrophobic surfaces in the parental and mutant strains. A 50 µm scale bar is shown in all pictures.

In both genetic backgrounds, the absence of BcHOX8 caused an obvious modification of fungal growth. When inoculated on commonly used solid media, mutant colonies appeared smaller and more compact than their parent counterparts. Radial growth of the two mutants colonies was 77–82% lower than that of their corresponding parents on TNK medium, 69–73% lower on PDA medium and 64–72% lower on malt sporulation medium (Fig. 2A). Microscopic observation of the colonies’ edges revealed higher branching frequency of the mutants hyphae when compared to the parent’s hyphae (Fig. 2B). When conidia in liquid PDB medium were deposited onto glass slides or Teflon membranes, the mutants hyphae grew in a multi-curved manner and formed arabesque-like patterns that were not observed in the parents samples (Fig. 2C). This curving effect could be seen soon after conidia germination, but could not be observed when germination took place in liquid medium only (data not shown). Since compact, slow-growth has already been compensated for by high osmolarity in B. cinerea [33], 0.2–1.0 M sorbitol or 0.1–0.8 M KCl was added to solid malt sporulation medium to test for possible compensation of the mutant growth defect. Compensation did not occur, but KCl had a positive impact on the mutants colony aspect (see below). Since restoration of growth defect by MgCl_2_ has also been reported [45], this salt was also tested, but no effect could be observed (data not shown). Based on the hyphal growth phenotype, the BcHOX8 mutants were renamed the Arabesque mutants.

### Impact of the BcHOX8 Mutation on Fungal Development

**Figure 3:**
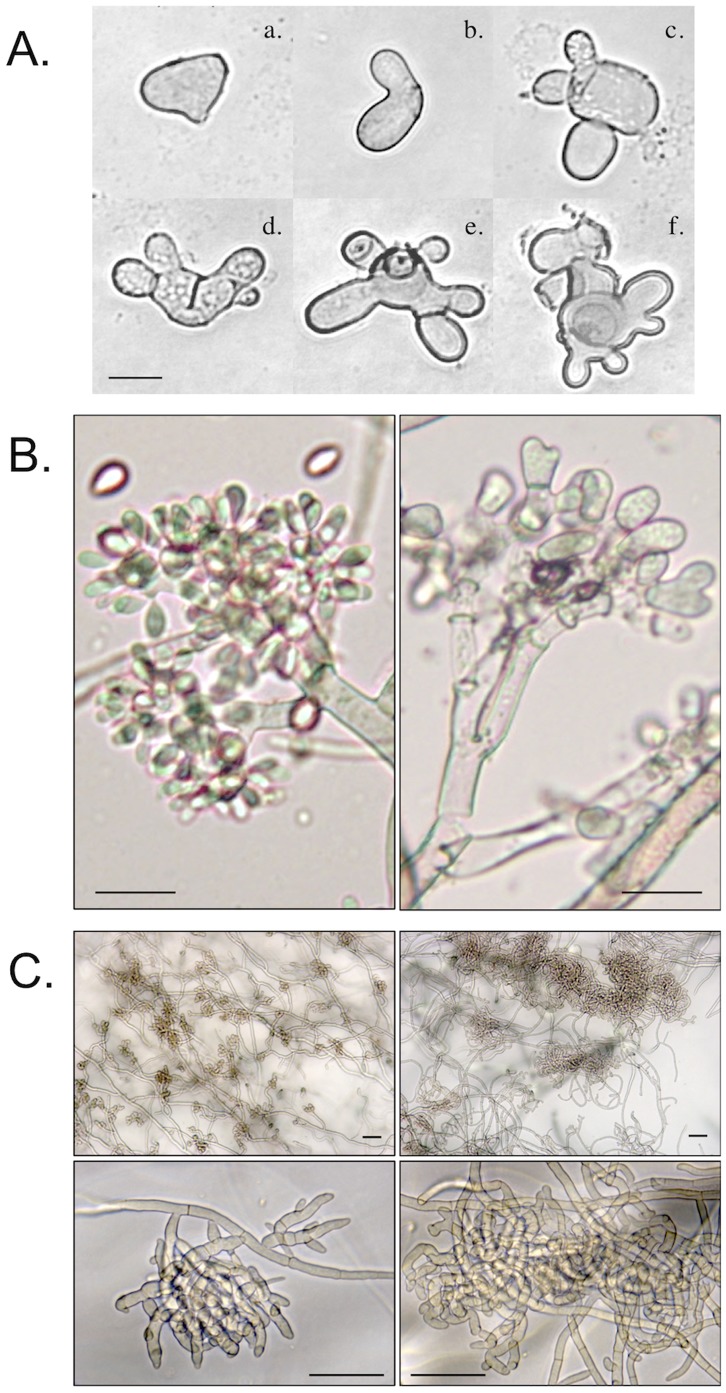
Impact of the BcHOX8 mutation on B. cinerea development. Microscopic observations of (A) various shapes of the Arabesque mutant conidia ranging from single (parent-like) to bi- (b: 24±6%), tri- (c: 8±3%) and multi-lobed (d–f: 20±5%) conidia, (B) conidiophores of the parental B05-10/Δku70 (left) and mutant B05-T_70-1_ (right) strains and (C) infection cushions in the parent B05-10/Δku70(left) and mutant B05-T_70-1_ (right) strains using 100x (C. top) and 400x (C. bottom) magnification. 10 µm, 25 µm and 50 µm scale bars are shown in panel A, B an C, respectively.

After 10 days of growth on rich medium under near-UV light conditions, B. cinerea colonies look homogeneously browny-grey and powdery as a consequence of conidiation. Under identical conditions, colonies of both the B05-T_70-1_ and T4-T_80-1_ Arabesque mutants appeared pale to greyish and exhibited patches of aerial hyphae. Collection and counting of the conidia showed a respective 98.4% and a 99.8% decrease in their production in the B05-T_70-1_ and T4-T_80-1_ Arabesque strains when compared to that of the parents (0.6–1 10^8^ conidia/plate), and this was not improved by longer culture times. Moreover, an average 42% of the Arabesque conidia exhibited abnormal morphologies (Fig. 3A) and so did the conidiophores (Fig. 3B). As described above, KCl had a positive impact on the colonies of both mutants. The latter resembled more that of their parents when the salt concentration was at least 0.6 M in the culture medium. This, however, did not relate to any restoration of the conidia production or morphology (data not shown) and MgCl_2_ also failed to improve either production or morphology.

The mutant conidia did germinate like their parent counterparts, and no difference in the production of sclerotia could be observed between the Arabesque and parent strains (not shown). The Arabesque mutants, however, produced large and abnormal infection cushions in vitro. Primary hooks in the mutant were numerous and appeared no different from their parental counterparts, but the finger-tip-like hyphae that constitute the infection cushions were interwoven in the Arabesque mutants instead of aligned in the parent strain, and this gave the infection cushions a disordered morphology (Fig. 3C).

### Impact of the BcHOX8 Mutation on Virulence

**Figure 4:**
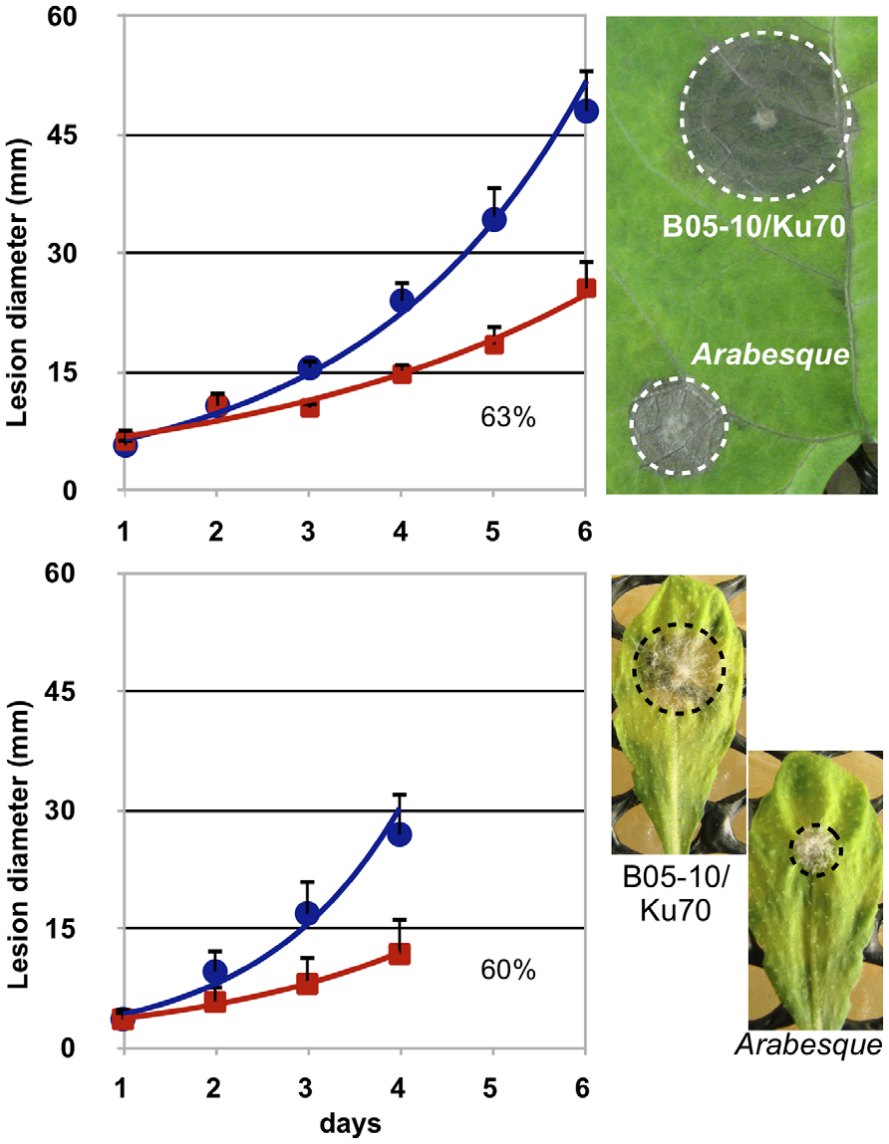
Impact of the BcHOX8 mutation on mycelium-derived infection. Kinetic of green bean (top) and thale cress (bottom) leaves infection by the parental B05-10/Δku70 (circle) and the Arabesque mutant B05-T_70-1_ (square) strains using 1.8 mm mycelium plugs as inoculum. In the case of green bean infection, 6 independent experiments using 6 leaves each (days 1 and 2) or 30 leaves each (days >2) were performed. In the case of thale cress infection, 3 independent experiments using 6 leaves each were performed. Monitoring of thale cress infection stopped when the necrosis zone of the parent control reached the leaves edges. Linearization of the exponential curves allowed calculation of the relative mutant growth when compared to its parent (% of the slope obtained for the parental strain). 4-days infection pictures are shown.

**Figure 5:**
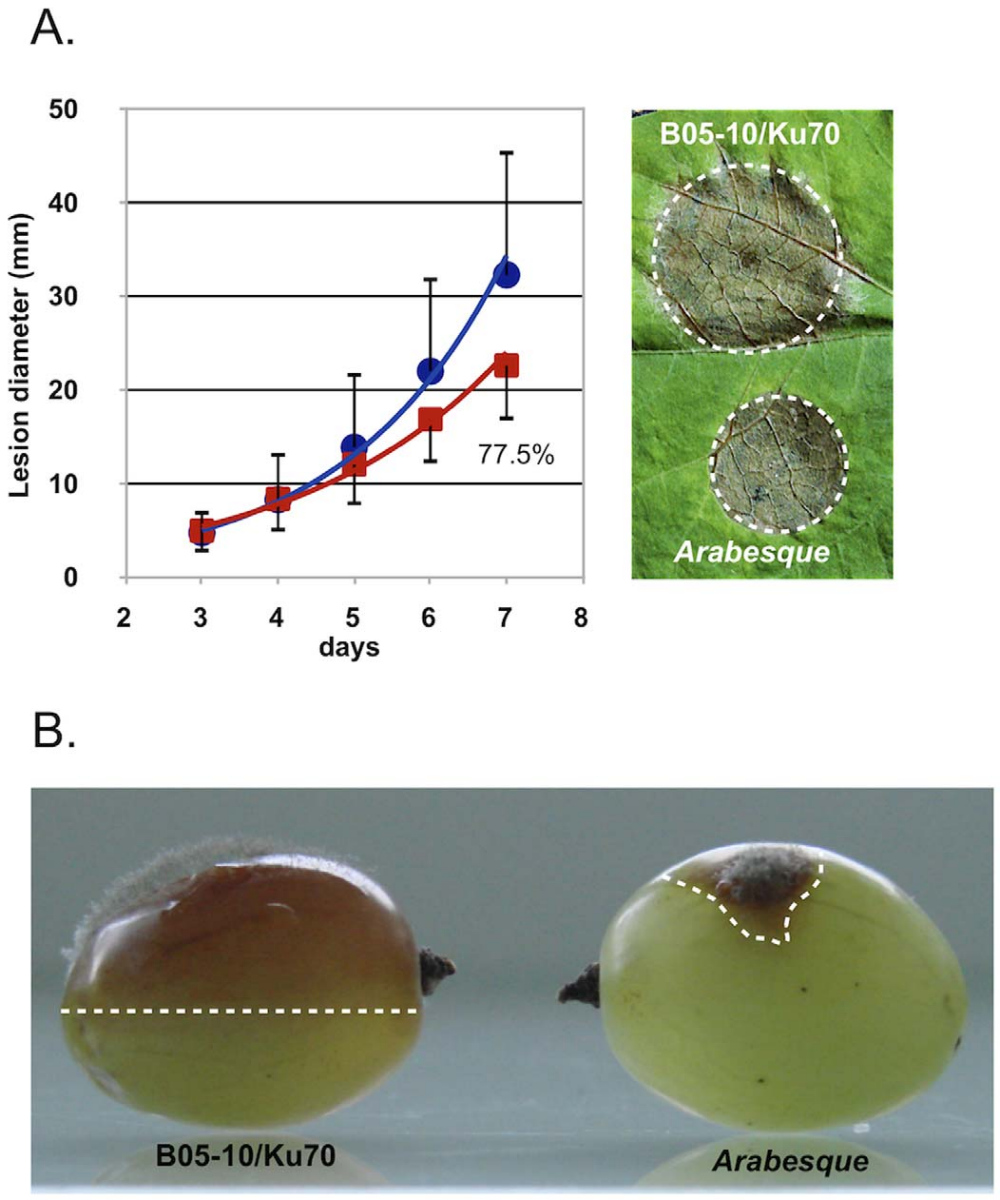
Impact of the BcHOX8 mutation on conidia-derived infection. Kinetic of green bean leaves infection by the parental B05-10/Δku70 (circle) and the Arabesque mutant B05-T_70-1_ (square) strains using conidia droplets as inoculum. Three independent experiments using 6 leaves each were performed. Linearization of the exponential curves allowed calculation of the relative mutant growth when compared to its parent (% of the slope obtained for the parental strain). A 7-days infection picture is shown. (B) Grape berries infection by the parental and Arabesque mutant strains using conidia as inoculum; a comparative 7-days infection picture is shown.

The impact of BcHOX8 deletion on virulence was next analyzed. Green bean or thale cress (A. thaliana)leaves infected with conidia or mycelium plugs collected from the Arabesque B05-T_70-1_ and T4-T_80-1_ strains exhibited smaller lesions than those infected by the parental strains, but these lesions eventually progressed throughout the entire surface (data not shown). In order to differentiate between an infection delay and a reduced invasion rate, B05-T_70-1_ and its parent were used in infection kinetics experiments. As shown in figure 4, necrosis could be observed at day 2 post-inoculation when parent or mutant mycelium plugs were used as inoculum. The tissue invasion then progressed exponentially, slightly slower on green bean than on thale cress leaves. On both host plants, the Arabesque’s progression did not appear, first delayed, and then identical to the parent’s counterpart, but exhibited instead a global 37–40% decreased rate, suggesting the absence of a plant penetration default in the mutant. When conidia were used as inoculum, necrosis and invasion were observed between day 3 and 4 post-inoculation for both strains. No specific further delay could be observed for Arabesque while its invasion rate appeared reduced by 22.5% (Fig. 5A); the high data variations however made the quantification uncertain. When grape berries, a natural host for conidia, were used instead of leaves, both a 5 fold reduction in the Arabesque capacity at causing any lesion and a slower progression inside the fruit (Fig. 5B) was observed when compared to its parent. To investigate further the putative impact of the mutation on plant penetration, infection kinetics were repeated using either mycelium plugs or conidia, and intact or wounded green bean leaves. Neither the mutant mycelium or conidia performed any better on wounded leaves (data not shown). Besides, onion epidermis penetration tests were used to microscopically monitor the early steps of infection via mycelium or conidia. Conidia from both the parent and mutant strains led to similar plant penetration rate and frequency. The only visible difference lied in the germ tube length produced prior to penetration, that of the mutant being longer (approximately 10 µm) than that of the parent (<5 µm). No difference could be observed between the parent and mutant samples when mycelium plugs or young mycelium grown in liquid medium were used. In both cases, infection cushions, penetration events and invasion of surrounding plant cells were seen (not shown). The morphology difference reported on Teflon membrane between the parent and mutant infection cushions was confirmed and one other qualitative difference was the propensity of the mutant invading hyphae to aggregate more parallel to each other in sometimes very dense patches. Altogether, these results indicate that BcHOX8 is not compulsory to plant infection by B. cinerea, but its absence is detrimental to efficient invasion of different host plants or tissues.

### Expression of the BcHOX8 Gene during Fungal Development

**Figure 6:**
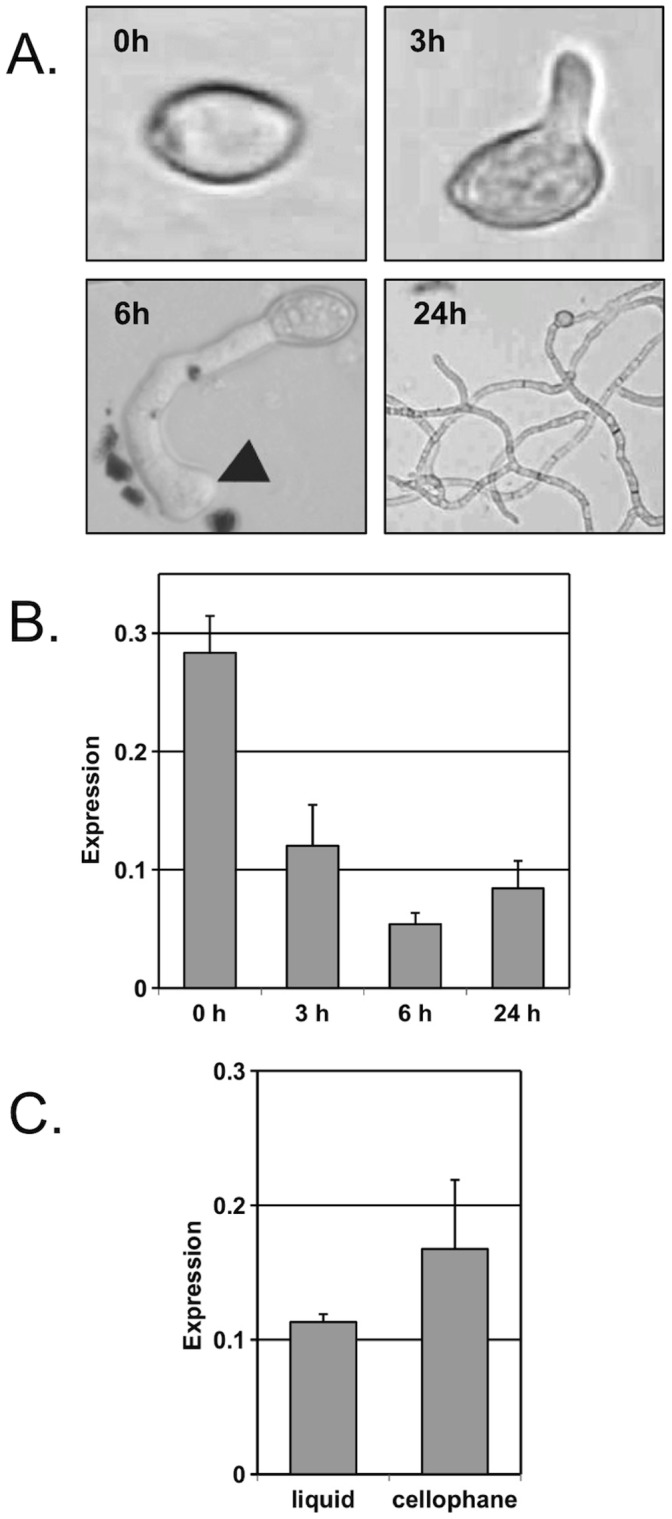
Expression of the BcHOX8 gene in the B05-10/Δku70 parental strain. (A) Kinetic of B. cinerea development on Teflon membrane using conidia as starting material; germinated conidia, appressoria (triangle) and young mycelium are shown. (B) BcHOX8 expression on Teflon. RNA were collected at the four stages shown in A and used for quantitative RT-PCR analysis (3 biological repeats). (C) BcHOX8 expression in infection cushions-enriched samples. RNA were extracted from mycelium grown in liquid cultures or onto floating cellophane (enriched in infection cushions) and used for quantitative RT-PCR analysis (4 biological repeats). Standard deviations are indicated and the actin-encoding gene was used as reference. When the EF1α-encoding gene was used as reference, identical results were obtained in B and a 2-fold higher differential was observed in C.

Whether the expression of the BcHOX8 gene would remain unchanged or vary during fungal developmental was assessed by measuring the production of its corresponding messenger RNA at different stages (Fig. 6A). Through deposition of conidia on Teflon membranes in diluted PDB medium, germinated conidia could be collected after 3 hours while material enriched in appressoria (curved and swelled germ tubes) could be harvested after 6 hours. In the absence of penetration through the artificial membrane, expanding young mycelium could then be obtained at 24 hours post-inoculation. Additionally, inoculation of cellophane sheets allows the collection of a fungal sample enriched with infection cushions (40%; J. Rollins, M. Choquer, personal communication) while hyphae can be collected from liquid cultures. Six distinct stages of the fungus development were hence collected from which RNA was extracted and analyzed by quantitative RT-PCR. When compared to the expression of the actin- or the EF1α-encoding genes, identical BcHOX8 expression profiles were monitored (Fig. 6B and C). Expression was high in conidia, dropped during germination and appressorium formation, increased slightly during mycelium formation on Teflon membrane and was higher in the infection cushions-enriched sample than in mycelium grown in liquid cultures. These results show that transcriptional modulation of the BcHOX8 gene occurs during the course of B. cinerea development.

## Discussion

The eukaryotic evolutionarily conserved homeobox genes are mostly known to regulate developmental differentiation. In fungi, mating has been shown to be under the control of such genes in several species, and examples are the ascomycete yeasts Saccharomyces cerevisiae, Schizosaccharomyces pombe and Candida albicans [70], the basidiomycetes Ustilago maydis, Cryptococcus neoformans, Coprinus cinereus and Phanerochaete chrysosporium [51], [53], [59], [71], or the filamentous ascomycetes *Cryphonectria parasitica*, *Neurospora crassa*, *Aspergillus nidulans*, *Sordaria macrospora* and *B. cinerea* [64]. Besides, homeobox genes were also shown to play a role in filamentous growth [51], [55], [60], hyphal morphology [58], [62], microconidia and conidia formation [58], [62], [63], appressorium formation [62], perithecium development [72] and clamp connections [73]. In addition, some homeobox genes were found to be important for virulence in the human pathogens C. albicans [74] and C. neoformans [75], and in the plant pathogens U. maydis [53] and M. grisea [62]. In this report, we describe the putative homeobox genes family of the plant pathogenic fungus B. cinerea and we present evidence that the BcHOX8 gene plays a role in vegetative growth and morphology.

According to the inventory made available for 58 pezizomycotina species on the fungal transcription factor database [65], all genomes except that of Sordaria macrospora harbour at least 6 predicted homeobox genes while less than 30% of them harbour 12 or more such genes. With 9 predicted BcHOX genes, B. cinerea holds a common position within this fungal group. Orthologues to all these nine genes were found in the genome of S. sclerotiorum, the closest relative B. cinerea, and no homologue to BcHOX4 and BcHOX5 have been found in other fungi but the latter, making these genes interestingly specific to these two plant necrotrophic pathogens. The absence of a homeodomain in the S. sclerotiorum SsHOX4 suggests however a likely functional divergence between these orthologues, or might explain biological differences between the two fungi once this gene will be characterized.

In the absence of functional data about the BcHOX genes and their orthologues in S. sclerotiorum, and based on the reported up-regulation of BcHOX8 during A. thaliana infection [65], we selected that gene as a first target for functional analysis. Through gene replacement in two different genetic backgrounds (B05-10 and T4 strains), the contribution of BcHOX8 to several aspects of B. cinerea life cycle was explored. Almost identical phenotypes were observed for the two mutant strains. Having verified by Sourthern that no ectopic integration of the DNA cassette occurred in the mutant strains, this strongly argues that these phenotypes are directly caused by BcHOX8 replacement, and not by some unknown genetic modification of the recipient strain following transformation. Indeed, the probability is very slim for two independent genetic events to occur in addition to the targeted gene replacement and to generate the same phenotype in two different protoplast populations, obtained from two different strains, and transformed independently. When compared to some instances of gene deletions that led to different phenotypes in different strains of B. cinerea [68]–[69], the almost identical phenotypes that we observed in the two mutant strains would moreover indicate that BcHOX8 plays a conserved function in B05-10 and T4 and that it is not influenced by these strains genetic backgrounds.

BcHOX8 deletion results in a slower radial growth rate and a higher branching frequency of the mycelium on solid medium. It also causes an unique curved pattern of hyphal growth on solid surfaces that led us to rename the BcHOX8 mutants the Arabesque mutants. It leads to very rare production of conidia and to their deformed morphologies. At last, it causes abnormal formation of infection cushions and reduces the fungus efficiency at colonizing plants tissues.

Branching in filamentous fungi is a phenomenon yet to be understood. It can be influenced by environmental factors like calcium [76], choline [77] or manganese [78] and can be modified by many mutations affecting cell signalling and transcription factors, cytoskeleton and associated motors, cell wall, ubiquitin-binding proteins, metal binding/metabolism proteins, reactive oxygen species control proteins or clock-related proteins [45], [79]–[86]. In addition to the Podospora anserina pah1 gene [58], BcHOX8 is the second homeobox gene whose mutation affects hyphal branching, and whether or how these two genes relates to one of the above functions needs to be clarified. Deletion of MoHOX1, the BcHOX8 homologue in M. oryzae, also causes growth rate reduction [62], but whether hyphal branching is modified has not been reported. Curving of the hyphal tips has not been reported either in this mutant or any other fungal homeobox gene mutant, and reasons for this polar growth alteration are unclear.

Conidiogenesis in B. cinerea is strongly affected by the absence of the calcineurin-responsive transcription factor BcCrz1, the MAP kinase BcBmp3 (homolog to the yeast SLT2 involved in cell wall integrity), the MAP kinase BcSak1 involved in osmoregulation, or the transcriptional regulator BcReg1, a downstream target of BcBmp3 and BcSak1 [45], [33], [31], [47]. Since the BcHOX8 mutation caused a severe diminution in conidia production, and since the proteins cited above are actors of signal transduction and regulation, the control of the BcHOX8 gene by these proteins might have explained the Arabesque phenotype. Differences between the latter and the phenotypes caused by all the above-cited gene deletions, however, argue against it: 1) Likewise BcHOX8 deletion, that of BcCrz1 affects hyphal morphology and their ability to colonize plants. However, Mg^2+^ restored growth and conidiogenesis of the Bccrz1 mutant and failed to do so in the Arabesque mutant. 2) In the Bcbmp3 mutant, sclerotia formation is lost and high osmolarity restores hyphal growth, but this is not observed in the Arabesque mutant. 3) The hyphal growth phenotype of the Arabesque mutant has not been reported for the Bcsak1 or the Bcreg1 mutants, and the misshaped conidia caused by the BcHOX8 mutation has not been reported in any of the other mutants mentioned here. Altogether, this would indicate that the BcHOX8 gene is a new transcription factor involved in conidiogenesis, and that it is not under the strict control of the gene products cited above. Finally, absence of the copper transporting ATPase BcCC2 also severely affects conidia production [23], and the control of the corresponding gene by BcHOX8 could hence be considered. If that were the case, however, the severe impact of BcCC2 deletion on melanization, sclerotia production and virulence should be observed in the Arabesque mutant, and it is not.

The Arabesque mutation does not prevent B. cinerea from infecting plants, as exemplified by the capacity of both the B05-10 and T4-derived mutants at colonizing green bean leaves, thale cress leaves or grape berries. On all host tissues used, penetration efficiency seemed not affected by the mutation, and this is supported by 1) similar microscopic observations of onion epidermis penetration by both the mutant and parental conidia or infection cushions, 2) the absence of delay in the mutant necrotic zone development on green bean and thale cress leaves when compared to that of the parent and 3) no difference between intact and wounded green bean leaves infections. On grape berries, however, mutant conidia failed to cause visible infection in 80% of the cases, and this might indicate some difficulty at penetrating harder epidermis. Once penetration has occurred, a clear impact of the Arabesque mutation can be seen on leaves or grape tissues colonization. Kinetic studies indeed demonstrated that the mutant progression in green bean or thale cress leaves was reduced 40% when mycelium was used as inoculum. When conidia were used instead, mutant progression inside grape berries was severely slowed down and a 23% reduction in leaves colonization was recorded (this last figure, however, can only be indicative due to high data variability). Taking into account the strong negative effect of the Arabesque mutation on hyphal growth in vitro (70–80% reduction), this decreased efficiency of colonization could be expected. It is nonetheless noteworthy that the mutant growth inside leaves tissues is better than the one measured in vitro on three different rich media; this might suggest the use of a specific growth pattern or nutrient metabolism during plant invasion whose dependence on BcHOX8 is weaker.

The BcHOX8 gene is differentially expressed during B. cinerea development in vitro. Expression is low in germinating conidia and vegetative mycelium, and higher in conidia or in infection cushions-enriched samples. The latter two structures appear misshaped when the BcHOX8 gene is absent, and it is therefore tempting to speculate a role for the BcHOX8 transcription factor in morphology control of these structures. In connection to this, expression of the BcHOX8 homologue MoHOX1 in the rice pathogen M. oryzae is specifically observed in mycelium [87] and deletion of that gene impairs fungal growth and melanin pigmentation, but not appressorium formation or conidial shape [62]. Based on macro-arrays and micro-arrays data, higher expression of the BcHOX8 gene only occurs at the early stage of A. thaliana infection [65], and does not change significantly during sunflower infection [40] or grape berries infection (A. Simon, personal communication). This would be consistent with the moderate impact of BcHOX8 deletion on fungal virulence and could suggest that the early peak of expression during A. thaliana infection is rather due to infection cushions formation than to plant invasion.

## Materials and Methods

### Bioinformatics

Predicted homeobox genes in *B. cinerea* were searched in the FTFD database (http://ftfd.snu.ac.kr). Blast and protein domains searches were performed using the Broad Institute database (http://www.broadinstitute.org/) and the URGI database (http://urgi.versailles.inra.fr/Species/Botrytis).

### Fungal Strains and Growth Conditions

*B. cinerea* strains were maintained on malt sporulation medium (malt extract 1.5%, glucose 0.5%, yeast extract 0.1%, tryptone 0.1% acid hydrolysate of casein 0.1%, ribonucleic acid 0.02%, 2% agar). The Petri dishes were inoculated with conidia (5×10^4^/plate) obtained from a 10-day-old culture and incubated at 21°C under near-UV light. The other media used for growth were TNK (glucose 1%, yeast extract 0,2%, NaNO_3_ 0,2%, KH_2_PO_4_ 0,2%, MgSO_4_ 7H_2_0 0,05%, CaCl_2_ 2H_2_O 0,01%, FeSO_4_ 7 H_2_O 0,0004%, microelements as in Tanaka B [88]), malt medium (malt extract 1%) and PDB (potato dextrose broth) or PDA (potato dextrose agar) (Difco). Mycelial samples enriched (40%) in infection cushions were prepared according to J. Rollins and M. Choquer (unpublished technique); Conidia in 1/4 diluted PDB (2×10^5^/plate) were spread onto dishes of 1/4 diluted PDA overlaid with cellophane and incubated for 44 h at 21°C.

### Construction of the Gene Replacement Cassette

The *BcHOX8* deletion cassette was obtained via double-joint PCR [89] in 3 steps: 1) Upstream and downstream regions of the *BcHOX8* gene were obtained by using genomic DNA (900 ng) as template, primers pairs fHox8up/rHox8up and fHox8down/rHox8down and 1 unit of iProof™ High-Fidelity DNA Polymerase (BioRad) in 200 µl reactions, 2) The NTC resistance cassette (nourseothricin acetyltransferase gene flanked by the *Aspergillus nidulans* OliC promoter and the *B. cinerea* Beta-tubulin terminator sequences) was amplified from plasmid pCB1003 using the primers pair fNour/rNour and 3) Amplification of the joint PCR products was obtained using the primers pair fHox8tot/rHox8tot. All primers are listed in Table S1 (supplementary material). The cassette was verified by DNA sequencing.

### Fungal Transformation

Young mycelium was harvested by filtration from a 16 hours liquid culture (6×20 ml) in malt medium at 21°C. Following wash (0.6 M KCl, 0.1 M phosphate buffer pH 5.8), it was suspended and agitated (50 rpm) for 3 hours at 21°C, in the same buffer containing Lysing Enzymes from Sigma (0.02 g/ml). Protoplasts were collected by filtration (nylon 30–40 µm), washed and suspended in cold KC (KCl 0.6 M, CaCl_2_-2H_2_0 50 mM, pH 6), and then incubated (2 10^7^/100 µl) for 20 min on ice with the deletion cassette (1–2 µg of PCR product) in 85 µl cold KC buffer plus 5 µl spermidine 0.075%. PEG4000 (25% PEG4000, 50 mM CaCl_2_-2H_2_O in 10 mM Tris buffer, pH 7.4) was added with gentle mixing and samples were then left for 30 min at room temperature. Following addition of 1.2 ml cold KC buffer, gentle mix and centrifugation (5 min, 5000 rpm), the protoplasts were suspended in 2 ml cold KC buffer, mixed (0.1 ml) with 10 ml 50°C SH medium (NaNO_3_ 1 mM, Sucrose 0.6 M in 5 mM TRIS buffer pH 6.5, agar Oxoïd 1.5%), poured into Petri dishes and incubated overnight at 21°C in the dark. The plates were finally overlaid with 10 ml SH medium containing 150 µg/ml nourseothricin (Werner BioAgents, Germany) and incubated at 21°C.

### Molecular Analysis of the Transformants

Following 2 days growth on malt sporulation medium overlaid with cellophane, mycelia were lyophilised. Genomic DNA was extracted (Qiagen Dneasy Plant mini kit) and used as template (200 ng in 50 µl reactions) for PCR amplifications with primers described in Table S1; hd1 and hd2 were used to amplify *BcHOX8* and Pr1, Pr2, Pr3 and Pr4 were used to amplify DNA fragments corresponding to the 5′ and 3′ cassette-locus junctions in transformants resulting from targeted gene replacement. For Southern hybridization, genomic DNA (5 µg) digested with *HindIII* was subjected to 1% agarose gel electrophoresis, transferred to positive nylon membrane (Q-BIOgene, Irvine, USA) and hybridized at 65°C in Denhardt’s buffer with a ^32^P-labelled probe. The radioactive signals were revealed using a phosphoimager (Molecular Dynamics, Sunnyvale, USA).

### Quantitative PCR Analysis

Experiments were performed essentially as in [90]. DNA-free total RNA (5 µg) served the synthesis of cDNA using Thermoscript RT (Invitrogen). qPCR reactions were performed using the Power SYBR® Green PCR Master Mix and an ABI PRISM 7900 HT from Applied Biosystems. Following examination of the primer efficiencies, DNA amplification was carried out as follows: 95°C for 10 min, 95°C for 15 s and 60°C for 1 min (50 cycles), 95°C for 15 s, 60°C for 15 s and 95°C for 15 s. Relative quantification was based on the 2^−ΔCt^ method using the actin- (accession number BC1G_08198) and ef1α- (accession number BC1G_04301) encoding genes from *B. cinerea* as references. Three independent biological replicates were analyzed.

### Phenotypic Analysis

Radial growth was measured daily following central inoculation of solid media with 50 µl of conidia suspension (10^5^/ml) and incubation at 22°C in the dark. Conidia production was measured by counting conidia under a microscope after collecting them from 10-days old cultures on malt sporulation media. Infection cushions formation was assayed through light microscopy after depositing 10^5^ spores/ml suspensions (in 1/4 diluted PDB) onto glass slides and their incubation at 22°C for 48 hours. Plant penetration and colonization were assayed through light microscopy after depositing conidia onto detached onion epidermis and incubation of the samples in humid chambers at 22°C for 24 and 48 hours, respectively. Infection assays on Arabidopsis thaliana (Col0 ecotype) and French green bean (Phaseolus vulgaris, Caruso cultivar) were performed with plants grown in a climatic chamber. Leaves were harvested and placed in a transparent plastic box lined with tissue moistened with water. Leaves were inoculated with 1.8 mm diameter-plugs of 3-days old mycelium obtained on solid malt medium or with 10 µl-droplets of conidial suspensions (10^5^ conidia/ml in 1/4 diluted PDB). Leaves in their boxes were incubated at 21°C under daylight exposure (16 h). Disease development was recorded daily as radial spreading from the inoculation point to the lesion margin. Pathogenicity assays were repeated as described in the figure legends. Infection assays on green grape (Vitis vinifera) were performed using a similar procedure on berries bought in a shop.

## Supporting Information

Table S1 **Primers used in the BcHOX8 study.**
(DOC)
