# Supplementary material for: The Homeobox BcHOX8 Gene in Botrytis Cinerea Regulates Vegetative Growth and Morphology
Source: PLoS One. 2012 Oct 25;7(10):e48134. doi: 10.1371/journal.pone.0048134 (PMC3485016; doi:10.1371/journal.pone.0048134)
Supplement: Table S1 — Primers used in the BcHOX8 study. (DOC) [file pone.0048134.s001.doc]

Table S1: primers used in the BcHOX8 study

| Primer name | Sequence |
| --- | --- |
| fHox8up | 5’-gcctagattacccgaagtgg- 3’ |
| rHox8up | 5’-gtaaagcctggggtgcct-cacgagtctctgtgatcgtc- 3’ |
| fHox8down | 5’-cttggctggagctagtggag-ccactctggacctttgttgg- 3’ |
| rHox8down | 5’-ttccgccaattctagccctt- 3’ |
| fNour | 5’-aggcaccccaggctttacac- 3’ |
| rNour | 5’-ctccactagctccagccaag- 3’ |
| fHox8tot | 5’-tgccacggcttatctttgtc- 3’ |
| rHox8tot | 5’-aggctcagctgcatattcag- 3’ |
| hd1 | 5’- ggacatgtcgcaagaagatg- 3’ |
| hd2 | 5’- cgaagttgttgttgctgctg- 3’ |
| pr1 | 5’-ctcgcatgggtgtaggtttt-3’ |
| pr2 | 5’-ggacgaaattggttcattcg- 3’ |
| pr3 | 5’-acgcaaatcttgaccaatcc- 3’ |
| pr4 | 5’-ctcgatctcgatctcgttcc- 3’ |
